# Resting State Functional Connectivity of the Rat Claustrum

**DOI:** 10.3389/fnana.2019.00022

**Published:** 2019-02-22

**Authors:** Samuel R. Krimmel, Houman Qadir, Natalie Hesselgrave, Michael G. White, David H. Reser, Brian N. Mathur, David A. Seminowicz

**Affiliations:** ^1^Center to Advance Chronic Pain Research, Department of Neural and Pain Sciences, School of Dentistry, University of Maryland, Baltimore, MD, United States; ^2^Department of Pharmacology, School of Medicine, University of Maryland, Baltimore, MD, United States; ^3^Department of Physiology, School of Medicine, University of Maryland, Baltimore, MD, United States; ^4^Graduate Entry Medicine Program, Monash Rural Health-Churchill, Churchill, VIC, Australia; ^5^Department of Physiology, Monash University, Clayton, VIC, Australia

**Keywords:** anterior cingulate cortex, caudate, cortex, forebrain, insula, putamen, striatum, top-down

## Abstract

The claustrum is structurally connected with many cortical areas.A major hurdle standing in the way of understanding claustrum function is the difficulty in assessing the global functional connectivity (FC) of this structure. The primary issues lie in the inability to isolate claustrum signal from the adjacent insular cortex (Ins), caudate/putamen (CPu), and endopiriform nucleus (Endo). To address this issue, we used (7T) fMRI in the rat and describe a novel analytic method to study claustrum without signal contamination from the surrounding structures. Using this approach, we acquired claustrum signal distinct from Ins, CPu, and Endo, and used this claustrum signal to determine whole brain resting state functional connectivity (RSFC). Claustrum RSFC was distinct from the adjacent structures and displayed extensive connections with sensory cortices and the cingulate cortex, consistent with known structural connectivity of the claustrum. These results suggest fMRI and improved analysis can be combined to accurately assay claustrum function.

## Introduction

The claustrum is highly interconnected with many cortical areas (Crick and Koch, 2005; Mathur, 2014). Early work in the cat suggests that the claustrum receives multimodal sensory input (Segundo and Machne, 1956; Spector et al., 1974). This concept was provisionally supported in a human study by Hadjikhani and Roland (1998) who showed that claustrum activation was greatest when both somatosensory and visual modalities were recruited in order to complete an object recognition task. In light of these data and taking the early view of Ettlinger and Wilson (1990) to a new conceptual level, Crick and Koch (2005) presented a new hypothetical framework: claustrum integrates multimodal information for the generation of conscious perception.

Countering the notion that the claustrum binds sensory information, later work in the cat (Olson and Graybiel, 1980; LeVay and Sherk, 1981) and monkey (Remedios et al., 2010) showed that only unimodal responses in the claustrum are detectable. Additionally, the anatomical pattern of claustrum connectivity shows that it weakly innervates primary sensorimotor cortices (White et al., 2017), but heavily innervates the medial prefrontal cortex (White et al., 2017) and anterior cingulate cortex (ACC) in marmoset (Reser et al., 2017) and rodent (Mathur et al., 2009; White et al., 2017, 2018). The ACC also projects heavily to claustrum (Smith and Alloway, 2010; White et al., 2017) and provides a top-down preparatory signal that is proportional to cognitive load (White et al., 2018). Moreover, ACC input to mouse claustrum innervates claustrum projection neurons that, in turn, project to parietal association cortex and visual cortices (White and Mathur, 2018). Together these data suggest that the claustrum may support top-down cognitive processing by coordinating multiple, widespread cortical regions. As such, ascertaining the global functional connectivity (FC) of the claustrum is required.

Resting state FC (RSFC) analysis as assessed with fMRI allows us to estimate region-to-whole-brain coupling (McIntosh, 2000). Given that the claustrum is a thin structure interposed between the insula (Ins), caudate/putamen (CPu), and endopiriform nucleus (Endo), it is likely that claustrum signal will have surrounding structures erroneously incorporated into it by virtue of partial effects, in which the signal from voxels within the claustrum also contain signals from tissue outside the claustrum boundaries (González Ballester et al., 2002; Du et al., 2014; Dukart and Bertolino, 2014). The goals of this experiment were to improve methodology used to study small volumes in fMRI and to reveal the RSFC of the claustrum in rats. We used rodent fMRI with a novel analytic technique called small region confound correction (SRCC) to isolate spontaneous claustrum signal from the Ins, CPu, and Endo to accomplish these goals. SRCC is an easy to implement method where “flanking” sections of structures adjacent to a small region of interest (ROI), like the claustrum, are regressed from the ROI, creating a confound-corrected ROI timeseries. Using this approach, we find claustrum is functionally connected with ACC, consistent with structural connectivity data (Reser et al., 2017; White et al., 2018) and that claustrum connectivity differs from the neighboring regions. Together these data set the stage for future analysis of claustrum participation in brain networks underlying cognition.

## Materials and Methods

### Animals

We used 10 adult female Sprague-Dawley rats (200–250 g, Charles River, DC, USA; 6 weeks) housed in ventilated plastic cages with soft bedding and kept on a 12/12 h light/dark cycle (lights on at 07:00), at constant temperature (22 ± 2°C) and humidity (50 ± 10%). Rats consumed standard rat chow and had *ad libitum* access to water. All procedures were approved by the Institutional Animal Care and Use Committee at the University of Maryland Baltimore and performed in accordance with the National Institutes of Health Guide for the Care and Use of Laboratory Animals.

### MRI Acquisition

MRI data were acquired with a Bruker BioSpec 70/30USR Avance III 7-Tesla scanner (Bruker Biospin MRI GmbH, Ettlingen, Germany) with a BGA12S gradient system and interfaced to a Bruker Paravision 5.1 console. A Bruker 40 mm circular polarized volume coil was used for data acquisition. During scanning, rats were anesthetized at a constant level of isoflurane (≤1.5%) and respiration and heart rate were monitored with a small animal monitoring and gating system and software (SA Instruments, Inc., Stony Brook, NY, USA). Anesthesia is well known to modulate the RSFC of the brain (Paasonen et al., 2018). These data were collected as part of a longitudinal study (see Hubbard et al., 2015), and subsequently we did not use terminal anesthetics like α-chloralose. Isoflurane may result in less localized brain mapping, so we used a low dose that mitigates these negative effects (Sommers et al., 2009; Williams et al., 2010). However, even low doses of isoflurane produce RSFC maps that differ from the awake brain more than other alternative anesthetics (Paasonen et al., 2018), and isoflurane suppresses network presence relative to medetomidine (Kalthoff et al., 2013). Throughout data acquisition, body temperature was kept at 36–37°C using a circulating warm water heater. A T2-weighted image was obtained (RARE, TR = 2,000 ms, TE = 14 ms, 256 × 256, in plane resolution = 100 μm, 24 axial slices, 1 mm slice thickness) for anatomical reference. Resting state scans were acquired using a spin-echo echo-planar imaging sequence (TR = 1,500 ms, TE = 35.0966 ms, 75 × 75, in plane resolution = 0.4 × 0.4mm, 1 mm slice thickness, 24 axial slices; 550 volumes).

### Preprocessing

All image preprocessing was conducted using SPM8^1^. The pipeline included slice timing correction, realignment, normalization, and smoothing with a 1 mm FWHM Gaussian kernel. We created a study-specific template by coregistering and averaging the T2-weighted images across animals and interpolating to voxel size of isotropic 0.5 mm.

### Region of Interest (ROI) Creation

**Figure 1:**
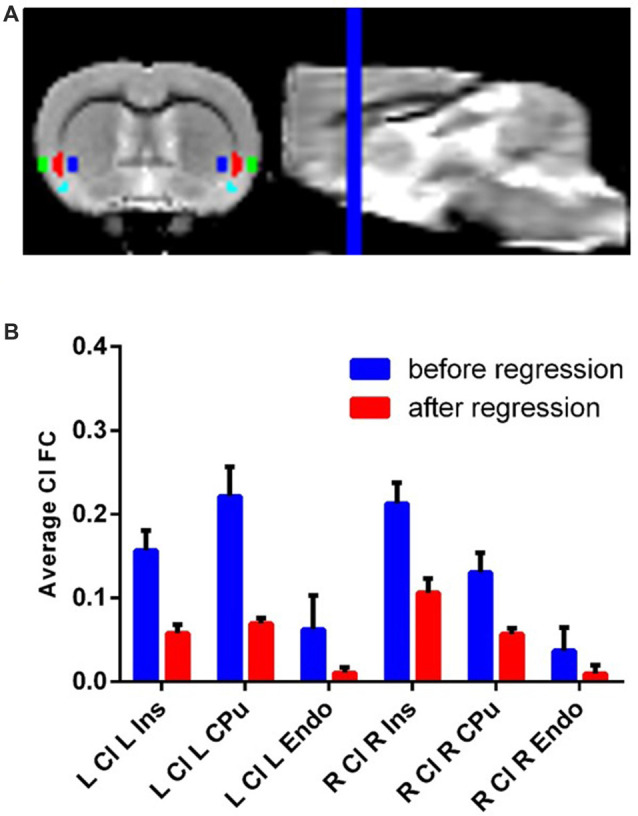
Claustrum signal independent of Ins, Cpu and Endo can be obtainedwith fMRI and a novel confound correction approach. **(A)** Structural MRI image with Ins (green), Cl (red), Endo (cyan) and Cpu (blue) on group template. These regions were hand-drawn on a group template made for this analysis. **(B)** Correlation (FC) of Cl timeseries with L/R Ins/Cpu/Endo before and after regressing out flanking regions. The average connectivity between Cl and its neighbors decreases to near zero following this approach. Error bars show standard error of the mean. Ins, Insula; Cpu, Caudate/Putamen; Endo, endopiriform; Cl, Claustrum; FC, Functional Connectivity; ROI, Region of Interest.

Each ROI (eight total: bilateral claustrum, Ins, CPu and Endo) was hand drawn on the previously described template brain resliced to 0.3 mm isotropic voxels (Figure 1A). We identified the claustrum based on known anatomical boundaries of the claustrum and used a parvalbumin staining from a separate dataset as a molecular marker for the claustrum (Qadir et al., 2018). The volume of each (i.e., left and right) claustrum was 2.86 mm^3^, or about 18 native resolution voxels. Additionally, we also drew a white matter and cerebro-spinal fluid (CSF) mask on this template.

### Small Region Confound Correction (SRCC)

fMRI suffers from partial volume effects, where voxels for the ROI contain tissues not belonging to the ROI (González Ballester et al., 2002; Du et al., 2014; Dukart and Bertolino, 2014). This creates ROI functional data with varying degrees of contamination from outside structures. This problem is especially pronounced for small structures like the claustrum, because the percentage of voxels with partial volume effects is quite high relative to very large structures. To address this problem, we treated sections of the major surrounding regions of the claustrum, the Ins, CPu, and Endo, as sources of noise, similar to how white matter is typically treated in resting state analysis. Specifically, we dilated the claustrum ROI by two voxels (0.6 mm) and created an overlap map with the Ins/Cpu/Endo and repeated the process with four dilations as well. Next, we subtracted the 2nd dilated overlap image from the 4th dilated overlap image. This created new “flanking” ROIs out of the medial section of Ins, lateral section of CPu, and dorsal section of Endo that roughly corresponded to the shape of the claustrum. We next treated these as sources of noise in RSFC analysis of the claustrum similar to white matter. This created claustrum FC maps that were linearly independent of partial volume effects from the Ins, CPu and Endo.

### Determining Functional Connectivity

Resting state preprocessing and seed-based analyses were conducted in the Conn toolbox version 17f^2^. Given continued controversy, we did not remove global signal from resting state data (Power et al., 2017). To account for noise present in white matter and CSF we used aCompCor (Muschelli et al., 2014) to determine the first five eigenvectors of white matter and CSF. We removed motion related signals estimated from the realignment parameters along with the first order derivatives of these parameters, in addition to the 10 previously mentioned eigenvectors for white matter and CSF. To avoid the reintroduction of noise while removing low frequency artifact, we used linear detrending and also simultaneously used a bandpass filter of 0.008–0.1 Hz. Despiking was used after these steps to remove any additional artifact that had yet to be removed (Patel et al., 2014). When determining claustrum connectivity, we also included the “flanking” segments of Ins, CPu, and Endo (see *SRCC*) as sources of noise. The timeseries for each ROI (excluding CSF and WM) was determined by averaging all voxels within the volume using *unsmoothed* functional data.

### Statistics and Data Availability

To determine significant RSFC, we performed one sample *t*-tests for resting state contrast maps with a cluster-forming threshold of *p* < 0.001 and an FWE cluster correction. This threshold has been shown to adequately control for false positive rates (Woo et al., 2014). To determine laterality of claustrum connectivity, paired *t*-tests for left and right claustrum resting state contrast maps with a cluster-forming threshold of *p* < 0.001 and an FWE cluster correction were performed. All data including code, ROI images, and the template brain are available upon request.

## Results

### Functionally Isolating the Claustrum From Surrounding Structures

Using resting state data, we observed timeseries correlation for: left claustrum left CPu *r* = 0.22, std = 0.11; left claustrum left Ins *r* = 0.16, std = 0.08; left claustrum left Endo *r* = 0.06, std = 0.13; right claustrum right CPu *r* = 0.13, std = 0.07; right claustrum right Ins *r* = 0.21, std = 0.08; right claustrum right Endo *r* = 0.04, std = 0.09. Following SRCC, we observed reduced timeseries correlation for: left claustrum left CPu *r* = 0.06, std = 0.03; left claustrum left Ins *r* = 0.07, std = 0.02; left claustrum left Endo *r* = 0.01, std = 0.02; right claustrum right CPu *r* = 0.06, std = 0.02; right claustrum right Ins *r* = 0.11, std = 0.05; right claustrum right Endo *r* = 0.01, std = 0.03 (Figure 1B). Thus, we were able to isolate claustrum signal from the surrounding regions using SRCC. To further validate this approach, we next applied this method to the ACC using bilateral M2 as surrounding regions and found that again, SRCC was able to isolate ROI signal (Supplementary Figure S1).

### Functional Connectivity of the Rat Claustrum

**Figure 2:**
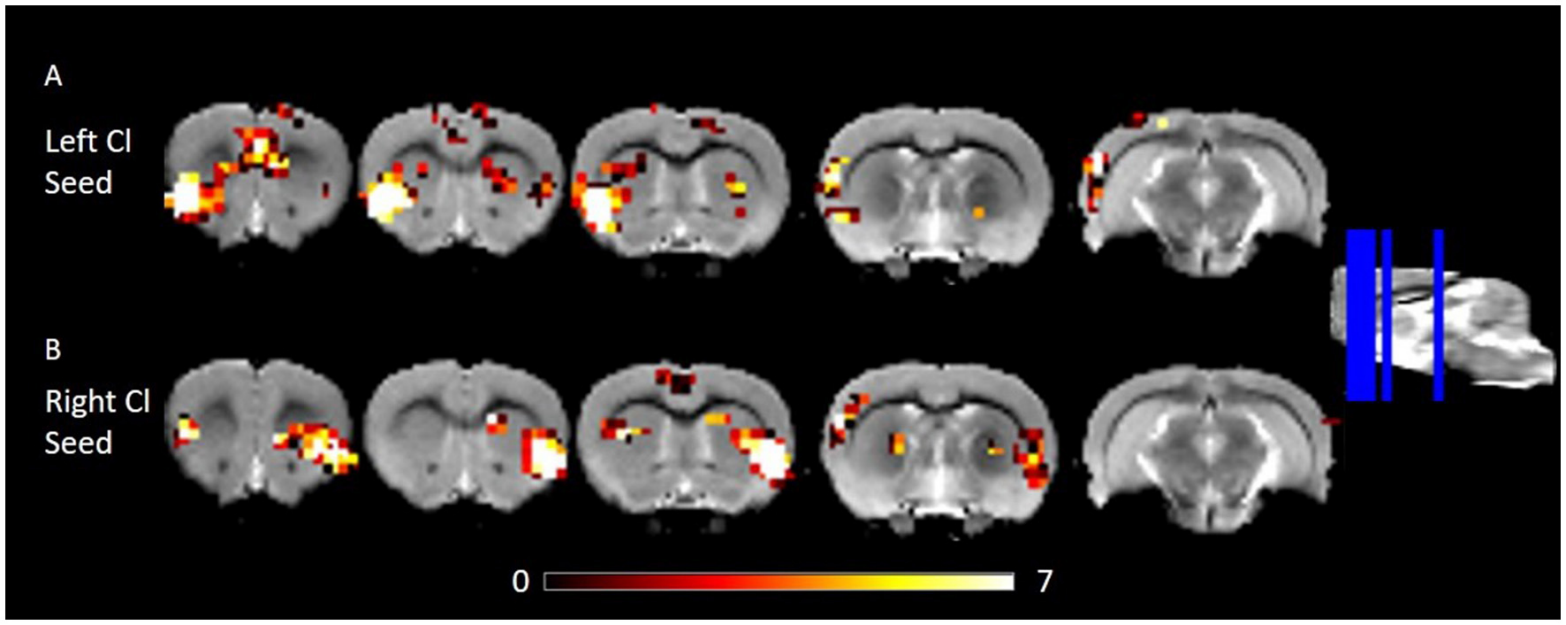
RSFC of the left and right Cl showing connectivity to frontal and posterior cortices. **(A)** RSFC of the left Cl. **(B)** RSFC of the right Cl. Data were thresholded at *p* < 0.001 followed by FWE cluster correction. Cl, claustrum; RSFC, resting state function connectivity.

We estimated FC of the corrected claustrum timeseries across the whole brain, anticipating that RSFC would be largely consistent with known anatomical connectivity of the claustrum. We therefore hypothesized that claustrum would have RSFC with sensory cortices and cingulate. Using the right claustrum seed, we observed strong RSFC with the bilateral cingulate, left claustrum, right auditory cortex, posterior area of the parietal cortex, visual cortex, S1, and S2 (Figure 2B). When using the left claustrum seed, we observed RSFC with the cingulate, CPu, auditory cortex, visual cortex, posterior area of the parietal cortex, S1, S2, and entorhinal cortex (Figure 2A).

### Laterality of the Rat Claustrum

**Figure 3:**
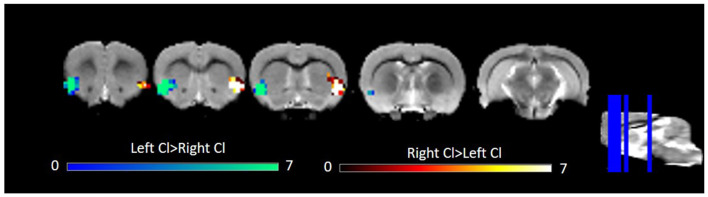
Laterality in RSFC of the left and right Cl. Data were thresholded at *p* < 0.001 followed by FWE cluster correction. Cl, claustrum; RSFC, resting state functional connectivity.

Several studies have observed laterality in claustrum activation (Hadjikhani and Roland, 1998; Naghavi et al., 2007; Lerner et al., 2008). Given this previous work and some qualitative differences in left and right claustrum RSFC maps in our data, notably left claustrum RSFC that spanned less of the cingulate than did RSFC of the right claustrum, we sought to quantitatively examine laterality. Using a paired *t*-test, we observed only bilateral clusters containing the claustrum (Figure 3). We did not find evidence for laterality in claustrum RSFC with the cingulate.

### Functional Connectivity of Claustrum vs. Surrounding Structures

**Figure 4:**
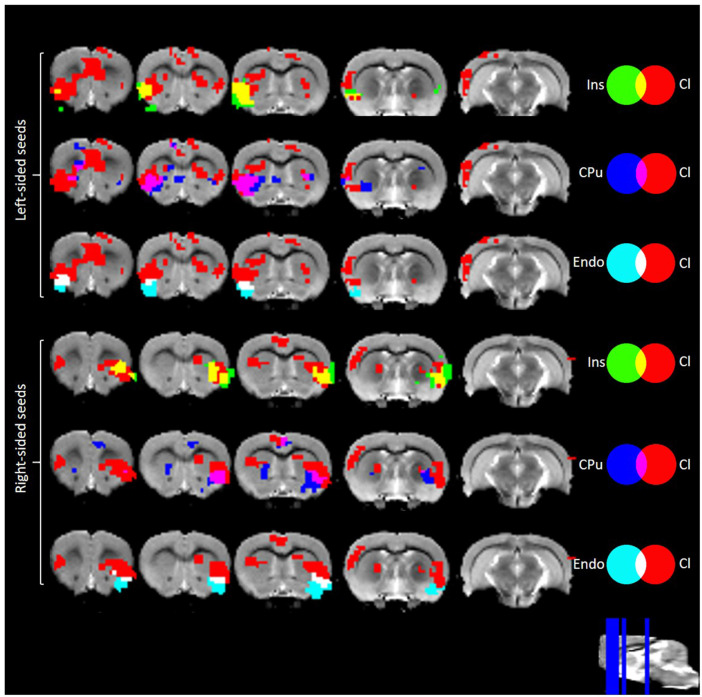
RSFC of Ins, Cpu, Endo, and Cl. These maps indicate a unique pattern of Cl connectivity relative to the surrounding structures. Data were thresholded at *p* < 0.001 followed by FWE cluster correction. Cl, claustrum; Ins, Insula; Cpu, Caudate/Putamen; Endo, Endopiriform; RSFC, resting sate functional connectivity.

Overlaying the RSFC maps of claustrum, Ins, CPu, and Endo bilaterally revealed distinct patterns of connectivity. Left claustrum displayed more extensive cingulate, S1, and auditory cortex RSFC than did the surrounding regions (Figure 4). The right claustrum displayed more extensive cingulate, S1, auditory cortex, and visual cortex than did the Ins, CPu, or Endo (Figure 4). Notably, the claustrum signal was not treated as a confounding source for the Ins/CPu/Endo. These data further support that we obtained a claustrum signal unique from the surrounding structures and are consistent with the extensive anatomical connectivity of claustrum to cingulate and sensory cortices.

## Discussion

In the current study, we propose a novel approach, SRCC, to study the claustrum using fMRI that overcomes existing problems in the field, namely, the masking of signal from an ROI by incorporating outside tissue into the ROI. This is an especially important problem in claustrum research because small volumes are particularly vulnerable to partial volume effects. Using fMRI and SRCC, we were able to isolate claustrum from Ins, CPu, and Endo, and show that claustrum displays robust RSFC with the cingulate and sensory cortices, consistent with anatomical connectivity.

RSFC is a complex measure that does not precisely mirror structural connectivity. However, RSFC is often consistent with structural connectivity (Greicius et al., 2009) and reflects anatomical features (Gordon et al., 2017). Consistent with a relationship between functional and structural connectivity, our RSFC maps derived from claustrum bear overlap with known anatomical connectivity. The rat claustrum sends dense inputs to the entire cingulate cortex (Wang et al., 2017; White et al., 2017) and the ACC, in particular, sends a dense input to the claustrum (Smith and Alloway, 2010; Wang et al., 2017; White et al., 2017). The claustrum also projects to the auditory and visual cortices (Wang et al., 2017; White et al., 2017; White and Mathur, 2018), the posterior area of the parietal cortex (Wang et al., 2017; White et al., 2017; White and Mathur, 2018), and S1 (Wang et al., 2017; White et al., 2017). Although direct connections between claustrum and the CPu are not clearly supported in the literature, the claustrum shares connectivity with cortical areas that are structurally connected with the CPu: the ACC projects to the dorsomedial aspect of the CPu (Voorn et al., 2004) and visual, auditory, somotasensory and motor cortices project to the dorsolateral aspect of the CPu (McGeorge and Faull, 1989). Taken together, these data support the notion that our claustrum RSFC data are valid.

Using resting state data, we observed minimal similarity between the timeseries of claustrum and Ins/CPu/Endo, indicating minor incorporation of the Ins, CPu, and Endo signal into the claustrum ROI. To further isolate claustrum signal we regressed flanking segments of Ins/CPu/Endo from the claustrum in a novel approach called SRCC. SRCC further reduced the timeseries similarity between claustrum and the neighboring regions to near zero, meaning that an independent claustrum signal was obtained. There are several limitations with this approach. First, this method assumes that any similarity shared by claustrum and adjacent structures is a partial volume effect, and hence, a confound. Any true FC between claustrum and Ins/CPu/Endo was therefore removed in our approach. In rats, there does not appear to be anatomical connectivity between the CPu and claustrum (Smith and Alloway, 2010), suggesting that CPu and claustrum should have low RSFC. Some connectivity between Ins and claustrum may exist in in rats (Lipowska et al., 2000; Qadir et al., 2018), which may suggest that some Ins-claustrum FC could have been suppressed with SRCC. Limited RSFC between the claustrum and endopiriform nucleus is also expected given that the endopiriform nucleus and claustrum have distinct anatomical connectivity patterns in mice (Watson et al., 2017; Qadir et al., 2018). An additional limitation of SRCC is regressing out these surrounding signals from claustrum could make an artifactual claustrum signal. However, our resting state analysis showed largely similar bilateral claustrum FC and was consistent with well documented anatomical connectivity, strongly suggesting that the corrected claustrum timeseries is physiological. Following SRCC, we still observe overlap between claustrum RSFC maps and Ins/CPu/Endo RSFC maps. This cannot be a result of partial volume effects from the bordering tissues, because there is no linear relationship between claustrum and Ins/CPu/Endo. These results likely reflect shared functional coupling between these structures and cortical targets. SRCC offers a simple-to-implement methodology that can greatly enhance confidence in small volume studies with fMRI, such as the claustrum, habenula, bed nucleus of the stria terminalis, thalamic association nuclei, and ventral pallidum. Additionally, the value of the approach will increase as the resolution of the fMRI data decreases, potentially making SRCC even more useful in standard resolution human fMRI datasets than in the current dataset.

The choice of our anesthetic is an additional limitation. By virtue of these data being collected as part of a longitudinal design (Hubbard et al., 2015), we were not able to use terminal anesthetics like α-chloralose. However, non-terminal anesthetics like metodimine (Nasrallah et al., 2014) have shown more similar RSFC to awake animals (Paasonen et al., 2018), and better network structure (Kalthoff et al., 2013) than isoflurane. Future work should experiment with better anesthetic protocols, that will likely result in more reliable and more widespread claustrum RSFC maps.

In conclusion, we show that in rat fMRI combined with SRCC, we can obtain claustrum signal independent of the surrounding structures. Using this claustrum signal, we observed RSFC with strong similarity to known anatomical connectivity of the claustrum. Together these data set the stage for future analysis of claustrum participation in brain networks underlying cognition.

## Author Contributions

DS, DR, and BM conceived the research. DS, SK, MW, and BM designed and performed the research. NH generated claustrum masks. DS, SK, MW, and BM analyzed data. DS, SK, HQ, and BM wrote the manuscript.

## Conflict of Interest Statement

The authors declare that the research was conducted in the absence of any commercial or financial relationships that could be construed as a potential conflict of interest.

## Footnotes

^1^ http://www.fil.ion.ucl.ac.uk/spm/

^2^ http://www.nitrc.org/projects/conn
