# Erratum: Resting state functional connectivity of the rat claustrum

**DOI:** 10.3389/fnana.2024.1454746

**Published:** 2024-07-03

**Authors:** Frontiers Production Office

**Affiliations:** Frontiers Media SA, Lausanne, Switzerland

**Keywords:** anterior cingulate cortex, caudate, cortex, forebrain, insula, putamen, striatum, top-down

Due to a production error, there was a mistake in Figure 2 as published. The panels “A: Left Cl Seed” and “B: Right Cl Seed” were not included in the figure. The corrected Figure 2 appears below. The publisher apologizes for this mistake.

**Figure 2:**
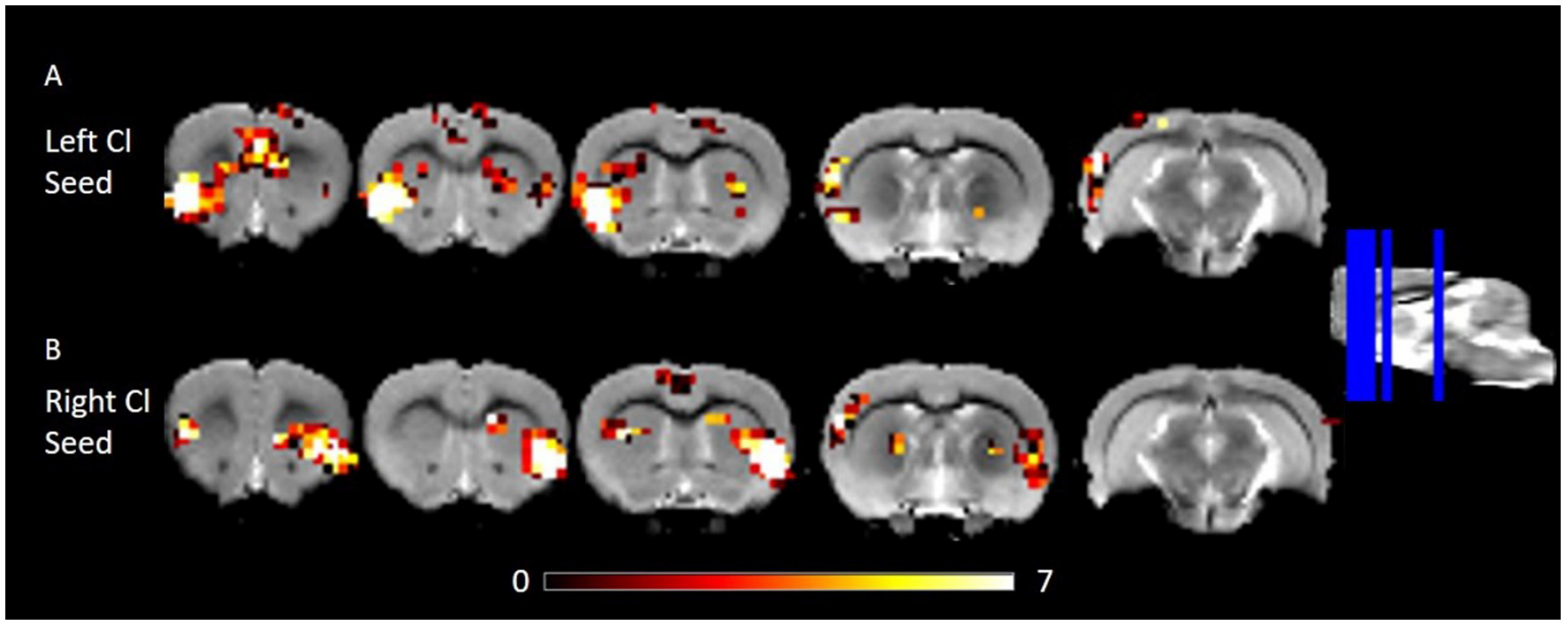
RSFC of the left and right Cl showing connectivity to frontal and posterior cortices. **(A)** RSFC of the left Cl. **(B)** RSFC of the right Cl. Data were thresholded at *p* < 0.001 followed by FWE cluster correction. Cl, claustrum; RSFC, resting state function connectivity.

The original article has been updated.

