# Supplementary figures and images for: Resting State Functional Connectivity of the Rat Claustrum
Source: Front Neuroanat. 2019 Feb 22;13:22. doi: 10.3389/fnana.2019.00022 (PMC6395398; doi:10.3389/fnana.2019.00022)

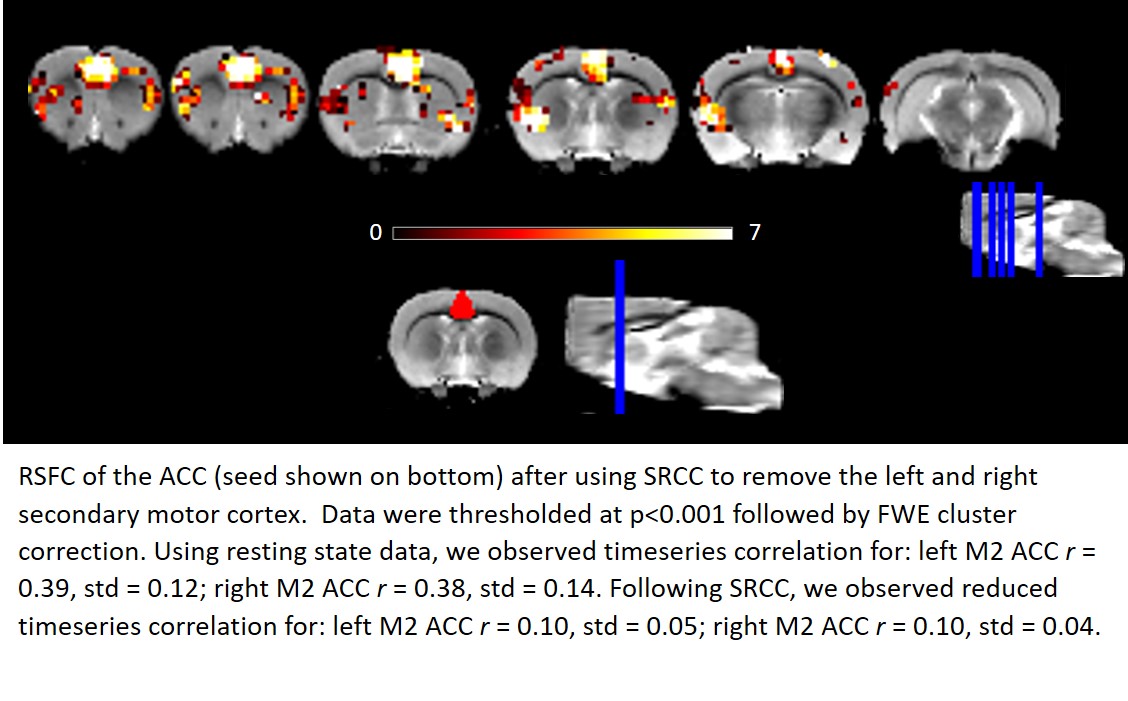

Supplement: Supplementary file 1 [file Image_1.jpg]
